# The effect of fast and slow decisions on risk taking

**DOI:** 10.1007/s11166-017-9252-4

**Published:** 2017-06-07

**Authors:** Michael Kirchler, David Andersson, Caroline Bonn, Magnus Johannesson, Erik Ø. Sørensen, Matthias Stefan, Gustav Tinghög, Daniel Västfjäll

**Affiliations:** 1 Department of Banking and Finance, University of Innsbruck, Universitätsstrasse 15, 6020 Innsbruck, Austria; 2 Centre for Finance, Department of Economics, University of Gothenburg, Box 600, SE-40530 Göteborg, Sweden; 3 Division of Economics, Department for Management and Engineering, Linköping University, SE-581 83 Linköping, Sweden; 4 Department of Economics, Stockholm School of Economics, Box 6501, SE-113 83 Stockholm, Sweden; 5 Department of Economics, NHH Norwegian School of Economics, Helleveien 30, NO-5045 Bergen, Norway; 6 The National Center for Priority Setting in Health Care, Department of Medical and Health Sciences, Linköping University, SE-581 83 Linköping, Sweden; 7 Department of Behavioural Sciences and Learning, Linköping University, SE-581 83 Linköping, Sweden; 8 Decision Research, 1201 Oak Street, Suite 200, Eugene, OR 97401 USA

**Keywords:** Prospect Theory, Experimental economics, Time pressure, Measurement noise, C91, C93, D81

## Abstract

We experimentally compare fast and slow decisions in a series of experiments on financial risk taking in three countries involving over 1700 subjects. To manipulate fast and slow decisions, subjects were randomly allocated to responding within 7 seconds (time pressure) or waiting for at least 7 or 20 seconds (time delay) before responding. To control for different effects of time pressure and time delay on measurement noise, we estimate separate parameters for noise and risk preferences within a random utility framework. We find that time pressure increases risk aversion for gains and risk taking for losses compared to time delay, implying that time pressure increases the reflection effect of Prospect Theory. The results for gains are weaker and less robust than the results for losses. We find no significant difference between time pressure and time delay for loss aversion (tested in only one of the experiments). Time delay also leads to less measurement noise than time pressure and unconstrained decisions, and appears to be an effective way of decreasing noise in experiments.

**Electronic supplementary material:**

The online version of this article (doi:10.1007/s11166-017-9252-4) contains supplementary material, which is available to authorized users.

## Introduction

Many economic and financial decisions appear to be taken rather automatically without much effortful reasoning. For instance, trading decisions on financial markets are taken within seconds after new information arrival (Busse and Green 2002). It is important to understand to what extent such fast decisions differ from more deliberative decisions. In this study, we experimentally test whether financial risk taking is systematically affected by the decision time available.

We implement a series of four experiments in three different countries on about 1700 subjects (a student population in Sweden, a general population sample in the US, and two student populations in Austria). Risk attitudes are elicited for both gains and losses by letting subjects choose between a sure gain (loss) or a 50% chance to win (lose) a larger amount. Subjects are randomly allocated to deciding within 7 seconds (time pressure) or waiting 7 or 20 seconds before deciding (time delay). The purpose of the experimental manipulation is to invoke relatively more intuitive decisions with time pressure and relatively more deliberative decisions with time delay.

Dual-process models are frequently used to explain differences between intuitive and deliberative decisions in psychology (Epstein 1994; Evans and Stanovich 2013; Glöckner and Witteman 2010; Kahneman 2003, 2011; Pham 2007; Stanovich and West 2000). In dual-process models, intuitive decision-making processes (System 1) are typically characterized as being fast, automatic, effortless, and emotional whereas deliberative decision-making processes (System 2) are characterized as being slower, more controlled, effortful, and deliberative (Epstein 1994; Kahneman 2003, 2011).^1^ Several different experimental manipulations have been used to invoke intuitive versus deliberative decision making. One of them is manipulating the timing of decisions (Finucane et al. 2000; Kocher and Sutter 2006; Kocher et al. 2013; Rand et al. 2012; Sutter et al. 2003; Young et al. 2012; Tinghög et al. 2013), which is the approach we use here. Other studies apply cognitive load or cognitive depletion tasks (Gilbert and Osborne 1989; Gilbert et al. 1995; Greene et al. 2008; Hagger et al. 2010; Shiv and Fedorikhin 1999; Schulz et al. 2014; Xu et al. 2012).

According to Kahneman (2011), the characteristics of System 1 are reflected in the features of choice behavior predicted by the S-shaped value function of Prospect Theory—risk aversion for gains, risk taking for losses (the reflection effect) and loss aversion. This interpretation is also supported by the results of Frederick (2005) who found that subjects who score low on the Cognitive Reflection Test (CRT) act more in line with Prospect Theory (more risk averse for gains and more risk seeking for losses) than high scorers. We therefore hypothesize that subjects (i) will be more risk averse for gains and (ii) more risk taking for losses with time pressure compared to time delay (i.e. we expect a larger reflection effect with time pressure than with time delay).^2^

We find that, on aggregate, subjects are significantly more risk averse for gains and more risk taking for losses in the time-pressure treatment than in the time-delay treatment. Moreover, we observe that the effect in the loss domain is significant in all four experiments separately. The effect in the gain domain, however, is slightly weaker and less robust. To control for different effects of time-pressure and time-delay on measurement noise, we estimate separate parameters for noise and risk preferences within a random utility framework. As expected, there is more measurement noise in the time-pressure treatment than in the time-delay treatment.

Following the argument made by Kahneman (2011) about the value function in Prospect Theory capturing the characteristics of System 1 decision making also implies that loss aversion should be higher with time pressure than with time delay.^3^ This is tested in one of the four experiments, but we cannot reject the null hypothesis of no difference across the two treatments. In one of the four experiments, we also include a treatment without time constraints. With this treatment, we test whether our results are primarily driven by forcing subjects to decide quickly or by forcing them to wait and think about their decision compared to unconstrained decisions. Our results for losses suggest that our treatment effect is driven by forcing subjects to take slow decisions rather than forcing them to respond fast. For gains, our results are inconclusive as the results for the time-pressure and time-delay treatments do not differ in the experiment with the unconstrained treatment.

As a related finding to ours, Porcelli and Delgado (2009) found that acute stress increased the reflection effect (increased risk aversion for gains and increased risk taking for losses). They argued that stress disrupts deliberative decision-making processes leading to System 1 thinking. In line with these findings, Cahlikova and Cingl (2017) stated that stress increased risk aversion for gains and Kandasamy et al. (2014) found similar effects when the stress hormone cortisol is administered exogenously.^4^

The most closely related study to ours is the recent paper by Kocher et al. (2013). They compared time-pressure with a no constraint treatment for pure gains, pure losses and mixed gambles. They found that time pressure decreased risk taking for losses, had no effect for gains and increased loss aversion (the mixed gambles). We find different results, although the comparability across the studies is limited. For instance, we only included a no constraint treatment in one of our experiments and they did not include a time-delay treatment. Furthermore, they collected data for two relatively small experiments (*n* = 176 and *n* = 95) and imposed a shorter time to respond in the time-pressure treatment (4 seconds rather than 7 seconds in our study). This may exacerbate problems with measurement error, and they do not separate out the effect of time pressure on risk taking from the effect of time pressure on measurement error.

## Experimental design

We carried out four separate experiments. In all four experiments, risky decisions for pure gain and pure loss prospects with real monetary stakes were included. In particular, we recruited 200 subjects from Linköping University in Sweden (Experiment SWE), 583 subjects from the population-representative subjects pool at Decision Research in Eugene, Oregon (Experiment USA), 320 subjects from the University of Innsbruck (Experiment AUT I), and 606 subjects in a second experiment at the University of Innsbruck (Experiment AUT II). In all four experiments, subjects were randomly allocated to the different experimental treatments. The four experiments are described in further detail below. The complete instructions of all experiments and screenshots of the decision situations can be found in the online appendix.

### Experiment SWE

The experiment was conducted at Linköping University in Sweden. Subjects were students at the Department of Management and Engineering, recruited through e-mail advertisement. Subjects did the survey in a computer lab, with no interaction allowed between individuals. The average sum paid out in the experiment was 130 SEK (around $19).^5^ The sessions were conducted between May and October 2012.

The experiment was part of a bigger data collection investigating the effect of time pressure on economic decision making. The complete survey was divided into six blocks: risk taking in the gain domain; risk taking in the loss domain; a public goods game; a dictator game; moral dilemmas and fairness judgments. To cover potential losses subjects were endowed with a show-up fee of 100 SEK.

In each session, subjects were randomly assigned to one of two treatments, the time-pressure or the time-delay treatment (TP and TD, respectively). Treatments were identical in all aspects, except that subjects in the time-pressure treatment had a maximum of 7 seconds to decide.^6^ A timer on the screen indicated how much time they had left to respond. Subjects in the time-delay treatment had unlimited time to respond, but were required to wait 7 seconds before any answer could be entered.

In the first risk taking block (in the gain domain), subjects made four sequential choices between winning a sum of money with certainty (option A) or to participate in a lottery (option B). The values for the safe option (A) increased from SEK 35 to SEK 50 and the lottery paid either SEK 0 or SEK 100 with equal probability. We did not provide subjects with a choice list, but presented each decision separately on the screen.

In the second risk taking block choices were identical, but in the loss domain. Subjects made four sequential choices between losing a sum of money with certainty (between SEK 35 and SEK 50) and participating in a lottery to lose either SEK 0 or SEK 100. A potential loss was covered by the show-up fee of SEK 100. The two risk taking blocks were the first two blocks that were played in each experiment. The block order was fixed, but the decision order within each block was randomized. Table 1 outlines the details on all risky decisions taken.

**Table 1:** Overview of the risk decisions in the four experiments (SWE, USA, AUT I, and AUT II)^a^

	**Option A: Safe amount**				**Option B: Amounts in 50–50 gamble**			
	SWE SEK	USA $	AUT I €	AUT II €	SWE SEK	USA $	AUT I €	AUT II €
Decisions: Gain Domain								
1	35	1.0	3.5	2.0	0/100	0/5	0/10	0/10
2	40	1.5	4.0	3.0	0/100	0/5	0/10	0/10
3	45	2.0	4.5	4.0	0/100	0/5	0/10	0/10
4	50	2.5	5.0	5.0	0/100	0/5	0/10	0/10
5				6.0				0/10
Decisions: Loss domain								
1	−35	−1.0	−3.5	−2.0	0/−100	0/−5	0/−10	0/−10
2	−40	−1.5	−4.0	−3.0	0/−100	0/−5	0/−10	0/−10
3	−45	−2.0	−4.5	−4.0	0/−100	0/−5	0/−10	0/−10
4	−50	−2.5	−5.0	−5.0	0/−100	0/−5	0/−10	0/−10
5				−6.0				0/−10
Decisions: Mixed gambles								
1				0				8/−2
2				0				8/−4
3				0				8/−6
4				0				8/−8
5				0				8/−10

^a^The order of the questions within each domain was random for each subject

All subjects decided anonymously and knew that everyone would answer the same questions. Before the session started, subjects were informed that 1 out of the 16 questions involving monetary payoffs would be randomly assigned for real payment in the end (questions in the block of moral dilemmas and fairness judgments were not incentivized). If a risk question was picked the subject either received the payout of the safe option or of the lottery according to her choice. Consequently, if a subject had chosen the lottery a coin flip was executed by the experimenter to determine whether she got SEK 0 or won (lost) SEK 100 for a question in the gain (loss) domain. If a risk question with a pure gain prospect was picked for real payment and the subject had not responded on time in the time-pressure condition, he received no payment from the question. If a risk question with pure loss prospect was picked for real payment and the subject had not responded on time, she received no show-up fee. This procedure was implemented in experiments SWE, USA and AUT I.

### Experiment USA

This experiment was run in collaboration with Decision Research in Eugene, Oregon and contained the same blocks as the first experiment (SWE). Subjects were drawn from a representative sample of the adult US population included in the subject pool of Decision Research. The experiment was conducted as a web survey. The same computer interface and design as in the first experiments was used. The design of the experiment was identical to the first experiment (SWE), with the only difference that the instructions were in English and stakes were smaller (see Table 1).^7^ The latter was necessary to ensure comparable stake sizes to other experiments at Decision Research. The average sum paid out in the experiment was 5.53 USD. The sessions were conducted during August 2012.

### Experiment AUT I

This experiment was conducted at Innsbruck ECONLAB at the University of Innsbruck in Austria. It contained the same blocks as the first experiment. Subjects were students from all faculties, recruited with ORSEE (Greiner 2004). Subjects did the survey in a computer lab, with no interaction allowed between individuals. The general structure of the survey was similar to SWE, but instructions were presented in German and stakes were in Euros (the stakes were similar to the ones in the first experiment; see Table 1). However, two changes were implemented. To ensure that subjects made deliberative responses in the time-delay treatment, we increased the time subjects had to wait before they were allowed to respond to 20 seconds. We also excluded the reminder sentence “Remember that you have a maximum of 7 seconds to answer each question” from the instructions in the time-pressure treatment. This was done to limit the possibility that subjects would prepare a calculated strategy for how to respond before seeing the actual questions under time pressure. The average sum paid out to subjects in the experiment was 12.35 Euros. The sessions were conducted in October 2012.

### Experiment AUT II

This experiment was conducted in Austria on a similar student sample as for Experiment AUT I (but no subject participated in both experiments) at Innsbruck ECONLAB at the University of Innsbruck in Austria. Subjects were students from all faculties, recruited with ORSEE (Greiner 2004). Subjects did the survey in a computer lab, with no interaction allowed between individuals. The other blocks included in addition to the risk taking blocks were not included in this experiment.^8^

As in Experiment AUT I, subjects had 7 seconds to reply in the time-pressure condition and had to wait 20 seconds before replying in the time-delay condition. The risk gain part and the risk loss part were identical to Experiment AUT I with the difference that the range of safe options was increased. The number of choices in the gain dimension was increased to five and the safe option varied between 2 and 6 Euros (see Table 1). For losses, the corresponding changes were made. We wanted to test if our results for gains and losses were robust to including a wider range of sure options, including one option with a sure gain (loss) larger than the expected gain (loss).

In this experiment, we added one more treatment in addition to time pressure and time delay. This third treatment did not involve any constraint concerning decision time and served to test whether an observed treatment effect is driven by responding under time pressure or having to wait before responding.

We also added a loss aversion block in this experiment to test if time pressure and time delay affects loss aversion. This part of the experiment comprised five questions. In these questions, individuals chose between taking a 50/50 gamble or not. In the 50/50 gamble, they could either win 8 Euros or lose between 2 and 10 Euros (see Table 1). The order of the 5 questions was randomly determined for each individual. The order of the 3 risk taking tasks was as follows: risk taking gains, risk taking losses and risk taking loss aversion (the mixed gambles).

After the three blocks testing for differences in risky decisions, the Jellybean task was conducted. The Jellybean task is used in psychology to test for intuitive versus deliberative decision making (Denes-Raj and Epstein 1994; Kirkpatrick and Epstein 1992; Peters et al. 2006). This task was therefore used to test whether the design of our experiment was successful in inducing intuitive and deliberative decision making. The instructions for the Jellybean task are included in online appendix A2.3. With time pressure subjects had to answer within 7 seconds and with time delay they had to wait 20 seconds before responding as below. In the no constraint treatment, no time constraint was imposed in the Jellybean task.

We furthermore included the Cognitive Reflection Task (Frederick 2005) in Experiment AUT II, to test if we could replicate the results of Frederick (2005). The CRT was included without any time constraint in all the three treatments.

The average sum paid out to subjects in the experiment was 10.6 Euros. The sessions were conducted in October and November 2013.

## Hypotheses

We hypothesize that time pressure leads to relatively more intuitive (System 1) decision making than time delay. Following the argument of Kahneman (2011) about System 1 being reflected in the S-shaped value function of Prospect Theory, we expect a stronger reflection effect (risk aversion for gains and risk taking for losses) and stronger loss aversion in the time pressure treatment compared to the time delay treatment. This leads to the following three hypotheses to be tested:

- Hypothesis 1: Time pressure leads to less risk taking than time delay in the gain domain.
- Hypothesis 2: Time pressure leads to more risk taking than time delay in the loss domain.
- Hypothesis 3: Time pressure leads to less risk taking than time delay for the mixed gambles.

Hypotheses 1 and 2 are tested in all four experiments, whereas Hypothesis 3 is tested only in Experiment AUT II. Note also that Hypotheses 1 and 2 can be thought of as a joint hypothesis for testing the reflection effect.

To test our hypotheses we will compare both the gambling rate and the estimated utility function between the time-delay and the time-pressure treatments. In the estimations in Section 5, we also separate out the effects of time pressure and time delay on risk preferences from their effects on measurement error.^9^

## Descriptive results

Table 2 provides descriptive results of the risk tasks. We calculate subjects’ gambling rates (the fraction of choices of the risky lottery B) in both treatments. In addition, we provide t-tests and Mann-Whitney U-tests for differences between treatments. For gains, the gambling rate is lower with time pressure compared to time delay for three out of the four experiments (although only marginally significant (10% level) in SWE and AUT I) which is consistent with Hypothesis 1. In the fourth experiment (AUT II), we find no significant difference between the two treatments.

**Table 2:** Descriptive statistics for the four experiments

	Treatment			*P*-value of TD vs TP^b^	
	TD	TP	NC	t-test	MW-test
**Experiment: SWE**					
GAIN: Gambling rate: % (SD)	73.02 (31.16)	64.63 (30.88)		0.0578	0.0254
LOSS: Gambling rate: % (SD)	34.65 (31.42)	43.28 (30.22)		0.0498	0.0451
GAIN: Missing responses (subjects): %^a^	0.00 (0.00)	3.83 (0.00)			
LOSS: Missing responses (subjects): %^a^	0.00 (0.00)	2.55 (0.00)			
N	101	98			
Women: %	45.00	41.24			
Mean age	23.30	22.39			
**Experiment: USA**					
GAIN: Gambling rate: % (SD)	78.75 (30.44)	68.95 (30.83)		0.0001	<0.0001
LOSS: Gambling rate: % (SD)	54.03 (38.93)	62.79 (37.28)		0.0057	0.0072
GAIN: Missing responses (subjects): %^a^	0.08 (0.00)	5.18 (0.00)			
LOSS: Missing responses (subjects): %^a^	0.00 (0.00)	3.77 (0.35)			
N	298	285			
Women: %	62.75	58.60			
Mean age	43.87	43.36			
**Experiment: AUT I**					
GAIN: Gambling rate: % (SD)	52.19 (35.29)	45.31 (34.79)		0.0802	0.0717
LOSS: Gambling rate: % (SD)	44.48 (32.50)	52.50 (34.45)		0.0329	0.0360
GAIN: Missing responses (subjects): %^a^	0.00 (0.00)	5.63 (0.00)			
LOSS: Missing responses (subjects): %^a^	0.03 (0.00)	2.50 (0.00)			
N	160	160			
Women: %	49.02	49.38			
Mean age	23.78	24.04			
**Experiment: AUT II**					
GAIN: Gambling rate: % (SD)	59.41 (21.94)	59.51 (26.35)	64.06 (22.86)	0.9671	0.7736
LOSS: Gambling rate: % (SD)	39.50 (19.59)	45.26 (24.10)	45.35 (22.55)	0.0091	0.0223
LOSS AVERSION: Gambling rate: % (SD)	49.31 (19.76)	51.51 (22.20)	48.61 (19.93)	0.2964	0.1624
GAIN: Missing responses (subjects): %^a^	0.00 (0.00)	5.54 (1.49)	0 (0.00)		
LOSS: Missing responses (subjects): %^a^	0.00 (0.00)	4.36 (1.49)	0 (0.00)		
LOSS AV.: Missing responses (subjects): %^a^	0.00 (0.00)	6.24 (2.48)	0 (0.00)		
N	202	202	202		
Women: %	56.93	57.43	59.90		
Mean age	23.53	23.48	23.43		
**Pooled Data: All experiments**					
GAIN: Gambling rate: % (SD)	67.27 (31.59)	60.75 (31.84)		<0.0001	<0.0001
LOSS: Gambling rate: % (SD)	45.59 (33.14)	53.28 (33.57)		<0.0001	<0.0001

^a^Missing responses are estimated as the fraction (%) of individual questions with missing responses. The number in parentheses gives the fraction (%) of individuals with missing responses on all 4 (5 in AUT II) gamble questions, who are not included in the gambling rate and statistical tests (i.e. all individuals with at least one response on the 4/5 gambling questions are included in the gambling rate and statistical tests)

^b^AUT II also included a no constraint (NC) treatment. The *p*-value of the t-test/MW-test is: 0.0375/0.0296 for TD vs NC and 0.0652/0.0926 for TP vs NC for GAIN; 0.0057/0.0180 for TD vs NC and 0.9703/0.9764 for TP vs NC for LOSS; 0.7258/0.9074 for TD vs NC and 0.1715/0.1382 for TP vs NC for LOSS AVERSION

In the loss domain, the gambling rate is significantly higher (at the 5% level) with time pressure than time delay in all four experiments which is consistent with Hypothesis 2. The no constraint treatment included only in the last experiment (AUT II) suggests that this effect is driven by the time-delay treatment rather than the time-pressure treatment.^10^

The analysis of the pooled data from all experiments shows that the difference is highly significant for both gains (*p* < 0.001) and losses (*p* < 0.001) in the hypothesized direction. The point estimate of the difference in the gambling rate in the pooled data is larger for losses (7.7 percentage units) than for gains (6.5 percentage units).

For the mixed gambles (loss aversion), which are only included in one of the experiments (AUT II), the gambling rate is very similar for the time-pressure and time-delay treatments and we cannot reject the null hypothesis of no effect.^11^

The fraction of missing responses among all choices is low and varies between 2.5% and 6.2% across blocks and experiments for the time-pressure treatment (in the time-delay and in the no constraint treatments the fraction of missing responses is close to zero; see Table 2). The rate of subjects in the time-pressure treatment that failed to respond to all questions within a block (and are thus missing in the significance tests and analyses) ranges between 0 and 2.5% across blocks and experiments. A low rate of missing responses is important as it minimizes a potential selection bias problem.

**Fig. 1:**
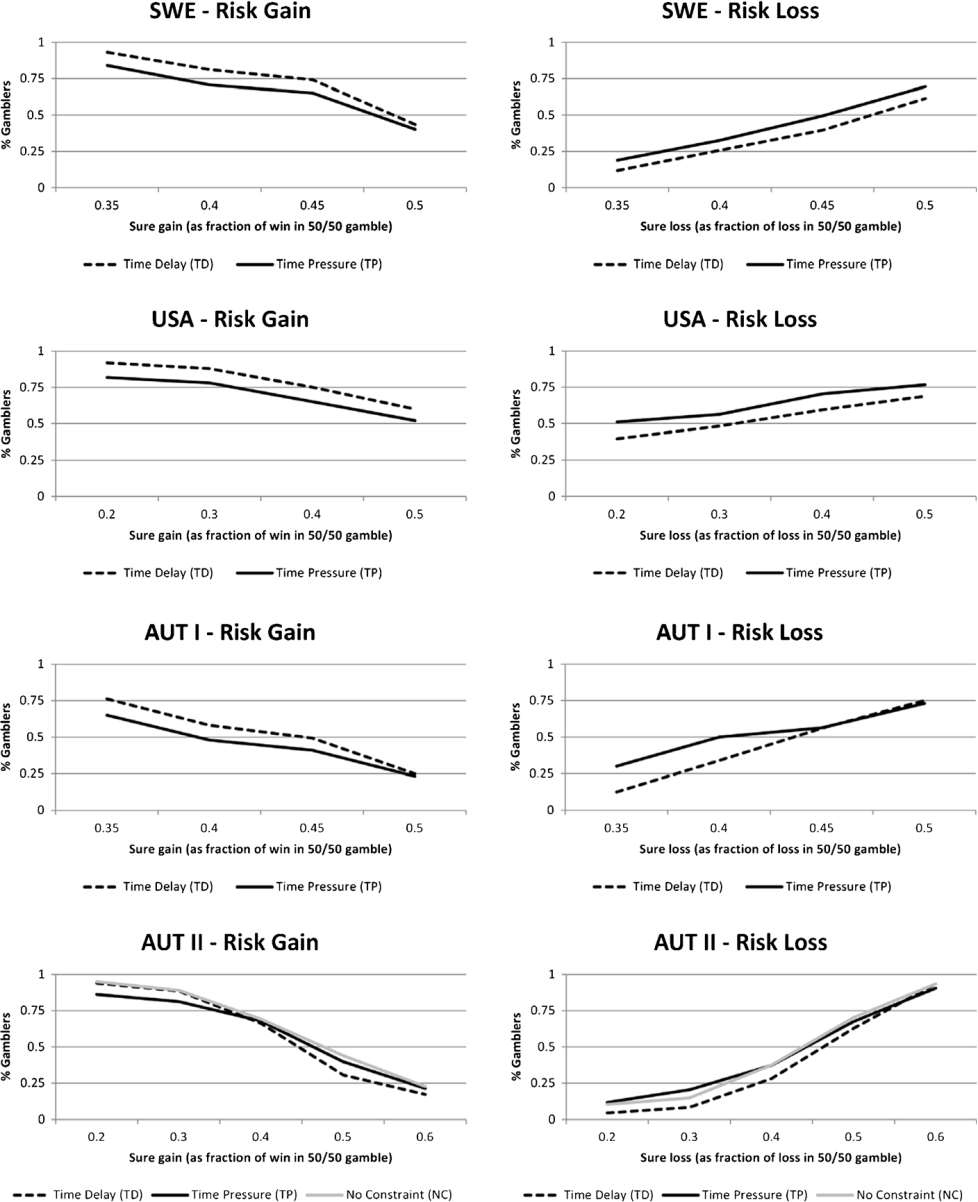
Percentage of decisions for the risky option B (the lottery) at each value of the safe option A (labelled in percent of the maximum potential gain/loss in the lottery)

**Fig. 2:**
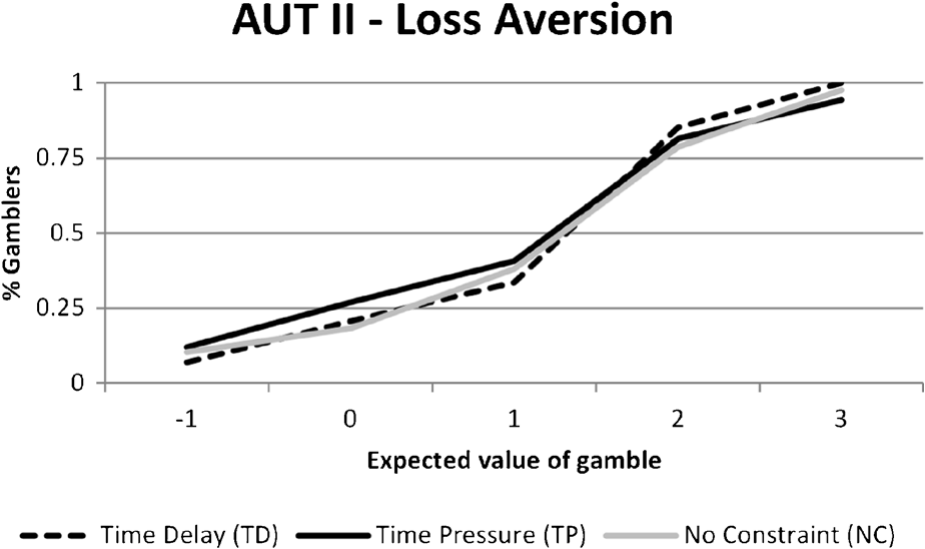
Percentage of subjects choosing the mixed gamble as a function of the expected value of the gamble

Figures 1 and 2 show the gambling rate as a function of the sure gain (loss) for the choices in the gain (loss) domain and the gambling rate as a function of the expected value of the gamble for the mixed lotteries. As expected, the fraction of subjects choosing the lottery declines as the value of the sure gain increases in the gain domain. In the three experiments SWE, AUT I, and AUT II the ratio of gamblers falls below 50% when the sure gain increases to the same expected value as the gamble (50% of the win in the gamble). This is evidence for risk-averse behavior in the gain domain as the certainty equivalent of the lottery is lower than the expected value for a majority of the subjects (an estimate of the median certainty equivalent in each experiment and treatment is where 50% of the subjects will gamble). In the US sample, the gambling rates are elevated and less than 50% of the sample is risk averse. This may be due to the lower stakes (Holt and Laury 2002). Time pressure shifts the gambling rates inwards compared to time delay in all samples except for AUT II.

Conversely, the fraction of subjects choosing the lottery increases as the value of the sure loss increases. This is evidence for risk-seeking behavior in the loss domain for a majority of subjects in all four experiments as more than 50% of the subjects choose the lottery when the sure loss equals the expected loss. In all samples time pressure shifts the gambling rates outwards compared to time delay.

For the mixed gambles (loss aversion) included in the last experiment, the fraction choosing to gamble as expected increases when the expected gain in the mixed gamble increases (Fig. 2). Less than 50% of subjects gamble when the expected gain is 0, consistent with a majority of the subjects being loss averse. However, there is no apparent systematic difference between the time-pressure and time-delay treatments.

In the AUT II experiment, we also collected data on the cognitive reflection task (CRT) to test if we could replicate the results of Frederick (2005). For the sake of comparability, we only use data in the no constraint treatment. Like Frederick we divide subjects into a low (0 correct answers; *n* = 58) and a high (3 correct answers; *n* = 31) CRT group. For losses we confirm the results of Frederick; the gambling rate for losses is significantly higher in the low CRT group (0.507 vs 0.381; *p* = 0.005). For gains we do not confirm the Frederick result, as the gambling rate does not differ significantly for the low and high CRT groups (0.631 vs 0.600; *p* = 0.575). The correlations between the number of correct CRT answers and the gambling rate also confirm these results (Pearson (Spearman) correlation for losses −0.171 (−0.145); *p* = 0.014 (*p* = 0.040); *n* = 202; Pearson (Spearman) correlation for gains −0.024 (−0.031); *p* = 0.735 (*p* = 0.664); *n* = 202). The gambling rate for the mixed gambles (not tested by Frederick) is not significantly correlated with the CRT answers (Pearson (Spearman) correlation −0.005 (0.011); *p* = 0.939 (*p* = 0.872); *n* = 202).

To test if our design is successful in inducing a difference in the degree of intuitive decision making, we consider the result of the Jellybean task in the last experiment (AUT II). With time pressure, 33.8% choose the larger bowl (i.e. the more intuitive choice), while only 18.3% choose the larger bowl in the time delay treatment. This effect is highly significant (*p*-value = 0.001, chi-square test, *n* = 344) and consistent with time pressure inducing more intuitive decision making than time delay.^12^^,^ 13 With no constraints, the fraction choosing the larger bowl is 28.2% suggesting that time pressure induces slightly more intuitive decision making than no constraints (*p*-value = 0.268, *n* = 344). Most importantly, time delay induces more deliberate decision making than no constraints (*p*-value = 0.018, *n* = 404), which indicates that our design is successful in inducing a difference in intuitive and deliberative decision making between the treatments.

The descriptive results overall support Hypotheses 1 and 2; but the support for Hypothesis 1 is weaker than the support for Hypotheses 2. Taken together the results for Hypotheses 1 and 2 support the joint hypothesis of an increased reflection effect in the time pressure treatment compared to the time delay treatment. For Hypothesis 3 we cannot reject the null hypothesis.

Apart from the observed risk-taking behavior, an important difference between the time pressure and the time delay treatment is the extent to which we observe inconsistent responses—i.e. subjects having more than one switching point. Depending on the experiment, 17% to 29% of subjects show inconsistencies in the time-pressure treatments, whereas only 3% to 15% do so in the time-delay treatments. This indicates that time pressure does not seem to affect behavior only through risk preferences, but also through increased noise in decision making. In the next section, we therefore separately estimate the effect of time pressure on risk attitudes and noise.

## Estimation results

Not only do we find time pressure to have a significant effect on risk-taking decisions, but we also see indications of time pressure leading to increased noisiness in behavior. To separate the effect time pressure has on risk preferences from the effect of time pressure on the degree of noise in decision-making, we estimate a model that allows disentangling these two underlying mechanisms.

In order to quantify effects on risk and loss aversion, we estimate the power utility function,

1 $$ u(z)=\left\{\begin{array}{c}{z}^{\gamma^{+}},\kern0.5em  z>0,\\ {}0,\kern0.5em  z=0,\\ {}-\lambda \cdot {\left(- z\right)}^{\gamma^{-}},\kern0.5em  z<0.\end{array}\right. $$

The specification of the utility function is the same one used by Tversky and Kahneman (1992) for Prospect Theory. The coefficient of risk aversion, *γ*, is allowed to differ for positive and negative amounts (hence the superscript) capturing the reference dependence of Prospect Theory. Prospect Theory assumes that the risk parameter is below 1 for both gains (risk aversion) and losses (risk loving).

The lambda parameter (*λ*) is the loss aversion parameter, and it is assumed to be larger than 1 in Prospect Theory (and equal to 1 in expected utility theory). The lambda parameter is only estimated in the mixed gambles included in the last experiment (AUT II).

We assume that choices are based on expected utility, and that a lottery *x* is valued *E*(*u*(*x*)) = ∑_*s*_ *p*_*s*_ *u*(*x*_*s*_). We thus do not incorporate the probability weighting part of Prospect Theory as all our gambles are 50/50 gambles. For estimation, we work with the certainty equivalent of the lottery, *V*(*x*) = *u* ^−1^(*E*(*u*(*x*))), since this parameterization allows a natural random utility application to choices over discrete lotteries (Von Gaudecker et al. 2011). For an individual who faces a set of lotteries X, let the random utility of each lottery $x\in \mathcal{X}$ be

2 $$ U(x)= V(x)+\xi \cdot {\varepsilon}_x, $$

with the standard assumption that the *ε*_*x*_s are independently and identically extreme value distributed. With the random utility assumption that choices maximize *U*(*x*) over X, probabilities of choices over lotteries now take the logit form (with a non-linear index). The likelihood contribution of an individual *i* choosing x from the choice set s = {x, y} is

3 $$ {\mathrm{L}}_{\mathrm{is}}=\Lambda \left(\left(\mathrm{V}\left(\mathrm{x};\upgamma, \uplambda \right)-\mathrm{V}\left(\mathrm{y};\upgamma, \uplambda \right)\right)/\upxi \right), $$

in which Λ is the standard logistic CDF. Estimation of the parameters, (γ, λ, ξ), is performed separately for each subsample using maximum likelihood, relying on the BFGS method that is part of the R library “stats4” (R Core Team 2014). Within each subsample, the parameters are modelled to be constant. Since the certainty equivalent *V*(*x*) is measured in currency units, *ξ* is identified, and estimates of *ξ* across different treatments allow comparisons of how large a role “noise” or randomness plays in a given treatment even if the curvature of the utility function is different. Throughout the paper, we refer to this as “measurement noise.” This definition does not refer to inaccuracies in recording the choices participants make, but rather to an element of randomness (noise) in the sequential choices made by subjects to elicit risk preferences. We convert all money amounts to US dollars so that estimates of *ξ* are comparable across countries.

First, we estimate the model on the pooled data (Table 3). For gains, the estimated parameter of risk aversion is 0.89 with time pressure and 0.92 with time delay, and this difference is significant. The estimated parameters for losses are 0.77 with time pressure and 0.85 with time delay. Again, this difference is significant. In summary, the estimated parameter for the pooled data is always closer to 1 with time delay, i.e. a behavior closer to risk-neutrality and money maximization. Furthermore, the estimated noise parameter is significantly higher for the time-pressure treatment than the time-delay treatment in both the gain domain and the loss domain.^14^

**Table 3:** Estimates of the utility function and the noise parameter for the pooled data across the four experiments^a^

	**Gains domain**			**Loss domain**		
Parameter	TP	TD	TP and TD	TP	TD	TP and TD
*γ*	0.887	0.924	0.908	0.769	0.848	0.812
	(0.012)	(0.009)	(0.007)	(0.011)	(0.010)	(0.007)
*ξ*	0.886	0.623	0.744	1.067	0.829	0.941
	(0.045)	(0.026)	(0.024)	(0.060)	(0.038)	(0.034)
TP vs TD:	*p*_*γ*_ = 0.014 , *p*_*ξ*_ < 0.001 , *p* < 0.001			*p*_*γ*_ < 0.001 , *p*_*ξ*_ < 0.001 , *p* < 0.001		

^a^All amounts are converted to US Dollars (using average exchange rates for 2013). γ denotes the coefficient of risk aversion, and ξ captures the measurement noise. The *p*-values reported are for *γ*, *ξ* , and for both parameters being the same in the TP and TD treatments. Inference for *ξ* is based on a parametrization in which log(*ξ*) = *μ*_0_ + *μ*_1_ *δ*_*TD*_, where *δ*_*TD*_ is a dummy indicator for a time delay session, and the reported *p*-value is for the restriction *μ*_1_ = 0. The joint tests are likelihood ratio tests. Standard errors are provided in parentheses

Taking into account country-level heterogeneity and minor implementation differences in the design of our experiments, we estimate the results for each of the four experiments separately (Table 4). For gains in SWE, the estimated parameter of the utility function (*γ* ^+^) is 0.94 with time pressure and 0.98 with time delay. This is consistent with Hypothesis 1, but the difference is not statistically significant. For USA, the coefficient of the utility function is 1.07 with time pressure and 1.19 with time delay. This effect, although not significant, also goes in the direction of less gambling with time pressure. Note, however, that the parameter of the utility function now implies risk loving behavior on average, which is inconsistent with Prospect Theory. This may be a consequence of the lower stakes in the USA data collection, encouraging more risk taking behavior and some utility in gambling per se. For AUT I the estimated parameter is 0.76 with time pressure and 0.83 with time delay, and this effect is statistically significant and consistent with Hypothesis 1. For AUT II, however, the coefficients are similar for time pressure and time delay and we cannot reject the null hypothesis of no difference. The coefficient for the no constraint treatment in AUT II is higher than for the time-pressure and time-delay treatments; but as there is no difference between these two treatments in AUT II it is not informative about which of these two treatments is driving the results in the gain domain in the other experiments.

**Table 4:** Estimates of the utility function and the noise parameter in the four experiments^a^

	**Gain domain**				**Loss domain**			
Parameter	TP	TD	NC	All	TP	TD	NC	All
**SWE:**								
*γ*	0.941	0.978		0.963	0.863	0.924		0.895
	(0.030)	(0.025)		(0.020)	(0.019)	(0.022)		(0.014)
*ξ*	1.183	0.844		1.010	1.017	0.964		1.000
	(0.195)	(0.115)		(0.107)	(0.144)	(0.133)		(0.099)
Tests, (TP vs TD):	*p*_*γ*_ = 0.338 , *p*_*ξ*_ = 0.116 , *p* = 0.010				*p*_*γ*_ = 0.036 , *p*_*ξ*_ = 0.784 , *p* = 0.044			
**US:**								
*γ*	1.072	1.190		1.151	0.436	0.589		0.514
	(0.074)	(0.071)		(0.054)	(0.034)	(0.025)		(0.020)
*ξ*	0.991	0.701		0.848	1.238	1.224		1.243
	(0.125)	(0.072)		(0.068)	(0.181)	(0.162)		(0.123)
Tests, (TP vs TD):	*p*_*γ*_ = 0.249 , *p*_*ξ*_ = 0.033 , *p* < 0.001				*p*_*γ*_ < 0.001 , *p*_*ξ*_ = 0.955 , *p* < 0.001			
**AUT I:**								
*γ*	0.763	0.826		0.797	0.790	0.844		0.822
	(0.017)	(0.014)		(0.010)	(0.016)	(0.011)		(0.009)
*ξ*	0.666	0.534		0.597	0.658	0.383		0.495
	(0.094)	(0.060)		(0.089)	(0.090)	(0.035)		(0.038)
Tests, (TP vs TD):	*p*_*γ*_ = 0.003 , *p*_*ξ*_ = 0.221 , *p* = 0.006				*p*_*γ*_ = 0.006 , *p*_*ξ*_ < 0.001 , *p* < 0.001			
**AUT II:** ^b^								
*γ*	0.892	0.873	0.948	0.903	0.811	0.880	0.811	0.835
	(0.023)	(0.017)	(0.021)	(0.011)	(0.017)	(0.015)	(0.015)	(0.009)
*ξ*	0.892	0.630	0.700	0.745	0.709	0.502	0.609	0.608
	(0.064)	(0.038)	(0.045)	(0.028)	(0.045)	(0.030)	(0.036)	(0.021)
Tests, (TP vs TD):	*p*_*γ*_ = 0.508 , *p*_*ξ*_ < 0.001 , *p* < 0.001				*p*_*γ*_ = 0.002 , *p*_*ξ*_ < 0.001 , *p* < 0.001			

^a^All amounts are converted to US Dollars (using average exchange rates for 2013). γ denotes the coefficient of risk aversion, and ξ captures the measurement noise. The *p*-values reported are for *γ*, *ξ* , and both parameters being the same in the TP and TD treatments. Inference for *ξ* is based on a parametrization in which log(*ξ*) = *μ*_0_ + *μ*_1_ *δ*_*TD*_, where *δ*_*TD*_ is a dummy indicator for a time delay session, and the *p*-value is for the restriction *μ*_1_ = 0. The joint tests are likelihood ratio tests. Standard errors are provided in parentheses

^b^The *p*-values comparing TP vs NC and TD vs NC are not shown in the table for lack of space. The *p*-values for the difference in the risk parameter (*γ*) between TP vs NC and TD vs NC are 0.071 and 0.005 for gains and 0.983 and <0.001 for losses. The *p*-values for the difference in the noise parameter (*ξ*) between TP vs NC and TD vs NC are 0.013 and 0.236 for gains and 0.082 and 0.022 for losses.

For losses, the estimated parameter of the utility function (*γ* ^−^) in SWE is 0.86 with time pressure and 0.92 with time delay, and this difference is statistically significant and consistent with Hypothesis 2. Note that a parameter of the utility function below 1 now implies risk taking behavior. The same is true for the US with a parameter of 0.44 for time pressure and 0.59 for time delay. The overall higher gambling rate in the USA data is evident for losses as well. For AUT I, the effect is also consistent with Hypothesis 2 and significant; the parameter is 0.79 with time pressure and 0.84 with time delay. In AUT II the estimated parameter is 0.81 with time pressure and 0.88 with time delay; again the effect is significant and in line with Hypothesis 2. The risk parameter for the no constraint treatment is 0.81, which is the same as for time pressure. This suggests that our treatment difference between time pressure and time delay in the loss domain is driven by the time-delay treatment leading to less risk taking compared to imposing no constraints.

In all of the experiments, the estimated noise parameter is higher for the time-pressure treatment than for the time-delay treatment, suggesting that the time-pressure treatment leads to more measurement noise. The difference in the estimated noise parameter is significant for two of the experiments in the gain domain and two of the experiments in the loss domain. The measurement noise parameter for the no constraint treatment in experiment AUT II is in between the other treatments. The *p*-values for the difference in the noise parameter (*ξ*) between TP vs NC and TD vs NC are 0.013 and 0.236 for gains and 0.082 and 0.022 for losses. To use a time delay procedure therefore seems to be an efficient way of reducing measurement noise in such experiments.

In the AUT II experiment, we also included decisions involving mixed gambles, allowing us to estimate a loss aversion parameter. To do so, since our variation in gambles is limited, we need to impose a restriction that risk aversion is the same in the gains and loss domain. Estimation results are shown in Table 5. In the top panel, we show estimates using only the mixed gambles. We see estimates of loss aversion around 1.4; this is consistent with a moderate degree of loss aversion in the sample, but the parameters are quite imprecisely estimated from the mixed gambles alone. In the lower panel of Table 5, we include the pure gains and pure loss gambles as well. This improves precision considerably, but does not change the level of estimated loss aversion. The quite small differences between treatments are not significant, and we conclude that there is no evidence of time pressure affecting loss aversion.^15^

**Table 5:** Estimates of the utility function, the noise, and the loss aversion parameters for AUT II^a^

Parameter	TP	TD	NC	All
**Estimated only on the mixed gambles in AUT II**				
*γ*	1.041	0.856	0.993	1.010
	(0.162)	(0.171)	(0.137)	(0.088)
*λ*	1.393	1.339	1.448	1.421
	(0.086)	(0.101)	(0.083)	(0.050)
*ξ*	0.802	0.354	0.632	0.651
	(0.247)	(0.215)	(0.208)	(0.132)
**Estimated on the full set of AUT II situations**				
*γ*	0.876	0.895	0.894	0.889
	(0.013)	(0.010)	(0.011)	(0.007)
*λ*	1.346	1.381	1.416	1.383
	(0.042)	(0.033)	(0.038)	(0.022)
*ξ*	0.722	0.517	0.611	0.613
	(0.030)	(0.019)	(0.023)	(0.014)

^a^γ denotes the coefficient of risk aversion, λ the loss aversion parameter and ξ captures the measurement noise. Estimated on AUT II data with a logit model as described in the main paper. Standard errors are provided in parentheses. Standard errors for *λ* and *ξ* are calculated with the delta method

Analyzing the data from the CRT in the AUT II experiment allows us to examine the heterogeneity in risk-taking behavior and the noise in individual decision making in more detail.^16^ We have interpreted the role of time pressure as provoking people to make more intuitive decisions, increasing the reflection effect and loss aversion. If so, we would also expect to see that individuals that are more prone to take intuitive decisions (measured by a low CRT score) would more often make such intuitive decisions within each treatment. To examine this, we parametrize both the risk aversion parameter *γ* and the multiplier on the random utility shock *ξ* as linear indices of the CRT score.

In Table 6, we see that both risk aversion and the noise parameter indeed are correlated with the CRT score. These results are weaker in the gain domain, where the only effect estimated to be large and significant is that high CRT individuals have much lower noise in the TD treatment. In the loss domain, the effects are more pronounced. In all treatments, higher CRT individuals are closer to risk neutrality and higher CRT individuals also have less noise in their decisions. These findings are in line with the non-parametric results reported in Section 4, and they corroborate the results of Frederick (2005). We find that scoring high in the cognitive reflection task is related to behavior being closer to risk-neutrality. In our case, this result holds in the loss domain, while in the gain domain, the average values for the risk aversion parameters are close to one, independently of the subjects’ CRT scores. That the CRT is related to the amount of noise in decisions is similar to the finding in Andersson et al. (2016b), who show that such noise is related to IQ, and that not properly accounting for this might lead to false inference with respect to risk aversion: Differences in the amount of noise might bias the estimate of risk aversion in either direction, depending on the choice architecture. We show, however, that even accounting for the effect on noise, there is still a correlation between CRT and risk aversion within treatment in the loss domain. We believe that the fact that there is such a strong relationship in the cross section, within treatment, lends some credence to our interpretation of the between-treatment differences.

**Table 6:** Heterogeneity analysis using CRT scores. Estimates of the utility function and the noise parameter for AUT II^a^

	**Gain domain**				**Loss domain**			
Parameter	TP	TD	NC	All	TP	TD	NC	All
*γ*_0_	0.932	0.847	0.975	0.917	0.766	0.824	0.754	0.782
	(0.039)	(0.030)	(0.038)	(0.020)	(0.027)	(0.024)	(0.026)	(0.015)
*γ*_*CRT*_	−0.028	0.019	−0.019	−0.010	0.032	0.041	0.042	0.038
	(0.019)	(0.015)	(0.021)	(0.011)	(0.015)	(0.013)	(0.014)	(0.008)
*μ*_0_	−0.035	−0.212	−0.234	−0.163	−0.186	−0.517	−0.163	−0.287
	(0.117)	(0.106)	(0.107)	(0.063)	(0.103)	(0.097)	(0.102)	(0.057)
*μ*_*CRT*_	−0.059	−0.195	−0.093	−0.108	−0.124	−0.148	−0.291	−0.175
	(0.063)	(0.059)	(0.061)	(0.034)	(0.055)	(0.057)	(0.059)	(0.032)

^a^γ denotes the coefficient of risk aversion. In this table both the coefficient of risk aversion and the logarithm of measurement noise coefficient are parametrized as linear indices of the cognitive reflection score, γ = *γ*_0_ + *γ*_*CRT*_^∗^ *CRT* and log(*ξ*) = *μ*_0_ + *μ*_*CRT*_^∗^ *CRT*. Estimated on the AUT II data with a logit model. Standard errors are provided in parentheses

## Conclusion and discussion

Overall, our results on risk taking provide support for Hypothesis 1 and 2 stating that time pressure leads to less risk taking in the gain domain and more risk taking in the loss domain compared to the time delay condition. The results are stronger and more robust in the loss domain, with a significant effect in the expected direction in all four experiments. In the gain domain, the results are weaker across the four experiments, but significant in the pooled data. Overall the results for Hypotheses 1 and 2 suggest that time pressure leads to a larger reflection effect compared to the time delay treatment. For Hypothesis 3 we cannot reject the null hypothesis, as we find no differences in risk taking between fast and slow decisions in mixed domain gambles. However, as we only collected data on mixed gambles in one of the experiments, we have less statistical power overall to detect an effect on risk taking in these situations.

The effect of time pressure on behavior might work through either risk preferences or increased noisiness in behavior, since participants engage in binary choices for which noisy behavior must lead to one-sided behavioral differences. To disentangle the role of risk preferences from that of noise, we estimate a choice model that allows these two mechanisms to have separate effects on outcomes. While we estimate there to be considerably more noise in behavior under time pressure in several of the treatments, under loss aversion we find that there are striking effects of time pressure also on risk preferences (above the effect on noise). In the mixed gambles in the AUT II experiment, we find no effect of time pressure on the estimated loss aversion parameter (while there are strong effects on the noise). In the AUT II experiment, we can also extend the choice model to examine within-treatment heterogeneity, allowing the participants’ CRT score to affect both risk aversion and noise. We see, again most clearly in the loss domain, that a high level of cognitive reflection is correlated with both less sensitivity to risk and less noise in behavior.

Although we cannot definitely identify the decision-making processes at work, our results are in line with the common finding that the intuitive System 1 is responsible for errors when making risky decisions, while System 2 mainly monitors the quality of the output of System 1 (Evans and Stanovich 2013; Kahneman 2011). Consistent with this, recent research has shown that individuals who are more capable in System 2 processing show less disposition to the reflection effect and more linear value functions (Schley and Peters 2014). One interpretation of the current findings is that time pressure decreases System 2 processing compared to time delay and thus increases the reflection effect. Following this logic, and as pointed out by Kahneman (2011), the S-shaped value function of Prospect Theory may primarily be a result of System 1 processing.

Our results are based on an extensive data collection involving over 1700 subjects in three countries, but further work is needed. It is not only important to accumulate more evidence on loss aversion, but also to acquire more evidence in the gain and the loss domains. This is also reinforced by the difference in our results and those of Kocher et al. (2013). Given the different setup of the studies, we identify the following features as the potentially most important drivers of the differences in results: The sample size and thus the statistical power are different as they collected data for two smaller experiments. A small sample size increases the risk of false positive results (Ioannidis 2005), and the importance of replicating results has recently been stressed in both economics and psychology (Maniadis et al. 2014; Open Science Collaboration 2015). Kocher et al. (2013) also use a more complex design, additionally varying the probabilities and impose more time pressure. Both a more complex design and having to respond in 4 seconds rather than 7 seconds may exacerbate the measurement error compared to our study. As we show in our study forcing subjects to respond fast increases the measurement error, and separating out the effect of time pressure on measurement error is important for testing whether time pressure affects preferences. The most robust finding across the two experiments in Kocher et al. (2013) is that they find a consistent and significant effect of time pressure on loss aversion in both of their experiments, which we do not find. As our test for loss aversion was only included in Experiment AUT II, it is possible that we fail to identify an effect in that direction that actually exists.

Also, subtle design differences and minimal changes in available decision time could be important in the time-pressure and time-delay treatments. Furthermore, our study highlights the importance of controlling for measurement noise in drawing inferences about the impact of time pressure and time delay on preferences. Our finding that a time delay procedure leads to less measurement noise than unconstrained decisions is important for experimental design in general. It implies that it may be beneficial in experiments to have a minimum waiting period before responses can be entered in order to increase the quality of the data.

Our results are potentially important for real-world decision making since most everyday decisions entail some degree of risk. For instance, traders on financial markets have to react to new information arrival within seconds. Decisions in bargaining tasks sometimes have to be taken within seconds and auctions usually end with a deadline. We especially believe that the increased risk taking behavior when deciding quickly in the loss domain is problematic. Thus, reducing time pressure and encouraging slower decision-making processes may be advisable whenever important (and costly) risky decisions have to be taken, especially when losses are involved.

## Electronic supplementary material

ESM 1 (PDF 949 kb)

## Footnotes

1 The distinction between System 1 and 2 has recently been popularized in the book “Thinking, Fast and Slow” by Kahneman (2011). Rubinstein (2007) has similarly argued that decisions in games that are made instinctively are faster than decisions based on cognitive reasoning. The dual-self model of Fudenberg and Levine (2006) proposes that many sorts of decision problems can be seen as a game between a long-run patient self and a sequence of short-run impulsive selves.

2 In the psychological literature on risk perceptions a distinction between risk-as-feelings and risk-as-analysis is sometimes made. Making decisions based on intuitive feelings is labeled risk-as-feelings, while following the rules of expected outcome and long-term consequences is referred to as risk-as-analysis (Loewenstein et al. 2001; Slovic et al. 2005; Slovic 2010). This can also be thought of as a System 1 and System 2 distinction, with risk-as-analysis referring to expected utility calculations.

3 This hypothesis is also in line with the recent findings of Andersson et al. (2016a) who found that loss aversion is lower in risk taking decisions on behalf of others. They argue that risk taking decisions on behalf of others are likely to be affected less by intuitive System 1 decision-making processes than risk taking decisions affecting the individual him or herself. See also the related paper by Pahlke et al. 2012.

4 There is also some psychological literature investigating time pressure in risky decisions. See, for instance, Ben and Breznitz (1981), Cella et al. (2007), Chandler and Pronin (2012), Verkoeijen and Bouwmeester (2014), and Young et al. (2012).

5 10 Swedish Kronor (SEK) were equivalent to 1.4 USD at the time of the experiment.

6 In the literature, the degree of time pressure imposed on subjects varies. For instance, Kocher et al. (2013) design a relatively strong time pressure regime by giving subjects only 4 seconds of time to respond. Rand et al. (2012) apply less pressure as subjects were able to decide within 10 seconds in their time-pressure condition.

7 A consequence of the lower stakes was also that the sure amount in the gamble varied over a somewhat larger range (as we wanted to avoid using two-place decimal numbers). In the first experiment (SWE) the sure amount varied between 35% and 50% of the potential win (loss) in the gamble, and in the second experiment (USA) the sure amount varied between 20% and 50% of the potential win (loss) in the gamble.

8 One difference compared to the first experiment in Austria (AUT I) was that z-Tree (Fischbacher 2007) was used to conduct the experiment rather than Qualtrics. This was done to handle the payments to the subjects more easily. We also implemented a slight modification with regards to the payment of subjects who failed to answer questions in the time-pressure treatment. If a question was picked for real payment and the subject had not responded on time she received no show-up fee in AUT II (no matter whether it was in the gain or in the loss domain). This was explicitly mentioned in the instructions. However, this modification had no significant effect on missing responses across the experiments. The number of missing responses in AUT II was even slightly higher compared to the other three experiments.

9 It should also be mentioned that differences in risk-taking decisions in the gain and loss domain might not only be the consequence of domain-specific differences in risk aversion. As pointed out by Vieider et al. (2016), changes in the parameters of the decision-situation could likewise lead to systematic differences in subjective probability weighting, while leaving the utility-curvature unaffected. With our design, we are not able to disentangle these two aspects and to identify a separate probability weighting effect as we do not vary the probabilities of prospects in our lotteries.

10 For the gain domain, the no constraint treatment is less informative as the time-pressure and time-delay treatments do not differ in the last experiment.

11 As robustness checks we run the same analyses and excluded subjects that did not answer all questions in the time-pressure condition. We find very similar effect sizes and *p*-values in the robustness checks.

12 Comparing data from the Jellybean task in the time pressure treatment to the other treatments, one has to take into account the fraction of missing observations in the time pressure treatment (30%, *n* = 60). These subjects failed to respond on time, and this could lead to a selection bias problem if late responders are more prone to select the smaller bowl. An upper bound on this bias is that all subjects that failed to respond on time would have chosen the smaller bowl, in which case the fraction choosing the larger bowl in the time pressure treatment would be 23.8%. This is still more than for the time delay treatment, but the difference is no longer significant (*p*-value = 0.179, chi-square test, *n* = 404).

13 Another caveat is that we do not control for any difference in measurement error between the treatments in the Jellybean task (as they only make one decision); so we cannot rule out that the observed differences are driven by differences in measurement error between the treatments.

14 Apesteguia and Ballester (2016) proposed that the profession estimate random preference models instead of random utility models. Random preference estimates are provided in Tables A1 and A2, and they lead to similar levels of estimated risk aversions as our estimated random utility models.

15 We show in Figure A1 in the online appendix that fixing the risk aversion anywhere in the range of [0.8, 1] has minimal effect on the estimated loss aversion parameter.

16 We thank an anonymous referee for this suggestion.
