# Supplementary material for: The effect of fast and slow decisions on risk taking
Source: J Risk Uncertain. 2017 Jun 7;54(1):37–59. doi: 10.1007/s11166-017-9252-4 (PMC5486903; doi:10.1007/s11166-017-9252-4)
Supplement: Supplementary file 1 — (PDF 949 kb) [file 11166_2017_9252_MOESM1_ESM.pdf]

# Appendix: The Effect of Fast and Slow Decisions on Risk Taking *

Michael Kirchler[1,2], David Andersson[3], Caroline Bonn[5], Magnus Johannesson[3,4], Erik Ø. Sørensen[5], Matthias Stefan[1], Gustav Tinghög[3,6], Daniel Västfjäll[7,8]

*Journal of Risk and Uncertainty 54(1)*

[*] We are grateful to Rudolf Kerschbamer, Marcus Mayorga, Shyam Sunder, Matthias Sutter, and Stefan Trautmann for very helpful comments on earlier versions of this paper. We thank conference participants at Experimental Finance 2013 in Tilburg and at the Time in Economic Decision Making Workshop 2014 in Munich for suggestions on improving the paper. Financial support by the Austrian Science Fund FWF (grant ZFP220400 Kirchler and START-grant Y617-G11 Kirchler), the Ragnar Söderberg, Jan Wallander and Tom Hedelius Foundation, and the Swedish Council for Working Life and Social Research is gratefully acknowledged.

[1] Department of Banking and Finance, University of Innsbruck, Universitätsstrasse 15, 6020 Innsbruck, Austria

[2] Centre for Finance, Department of Economics, University of Gothenburg, Box 600, SE-40530 Göteborg, Sweden

[3] Division of Economics, Department for Management and Engineering, Linköping University, SE-581 83 Linköping, Sweden

[4] Department of Economics, Stockholm School of Economics, Box 6501, SE-113 83 Stockholm, Sweden

[5] Department of Economics, NHH Norwegian School of Economics, Helleveien 30, NO-5045 Bergen, Norway

[6] The National Center for Priority Setting in Health Care, Department of Medical and Health Sciences, Linköping University, SE-581 83 Linköping, Sweden

[7] Department of Behavioural Sciences and Learning, Linköping University, SE-581 83 Linköping, Sweden

[8] Decision Research, 1201 Oak Street, Suite 200, Eugene, Oregon 97401, USA

## A1. Additional figures and estimations

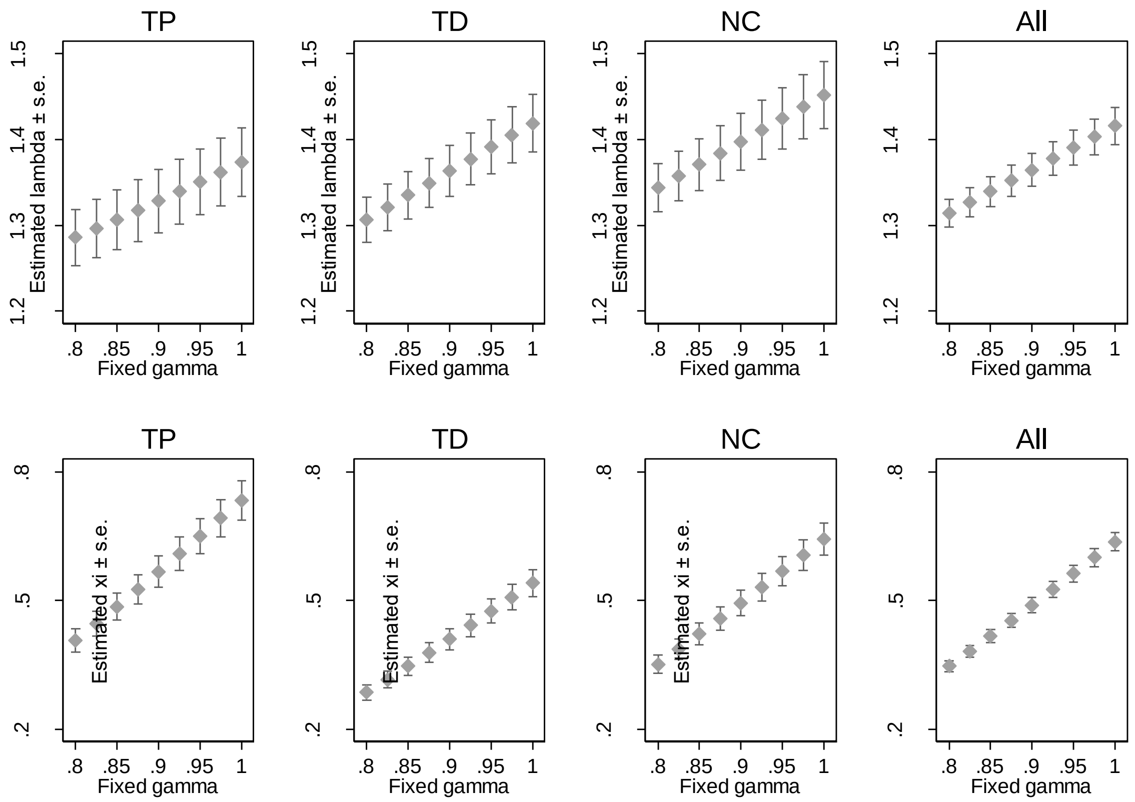


Figure A1. Estimates of the loss aversion parameter ($\lambda$) and the noise parameter ($\xi$) given fixed levels of risk aversion in the AUT II experiment. The graph shows the estimate and standard error bars for $\lambda$ (top panel) and $\xi$ (lower panel) in the AUT II experiment holding $\gamma$ constant at varying values on the interval [0.8,1.0].

Table A1: Different stochastic specifications (random preferences) for estimates of the utility function for the pooled data across the four experiments.[1]

| | **Gain domain** | | | **Loss domain** | | |
|---|---|---|---|---|---|---|
| Parameter | TP | TD | TP and TD | TP | TD | TP and TD |
| $\mu_\gamma$ | 0.915 | 0.955 | 0.938 | 0.727 | 0.827 | 0.783 |
| | (0.013) | (0.010) | (0.008) | (0.013) | (0.011) | (0.008) |
| $\sigma_\gamma$ | 0.471 | 0.352 | 0.407 | 0.533 | 0.440 | 0.483 |
| | (0.024) | (0.014) | (0.013) | (0.030) | (0.020) | (0.017) |

[1] This table corresponds to Table 3 in the main paper. All amounts are converted to US Dollars (using average exchange rates for 2013). We present estimates of "random preference" models in the sense of Loomes and Sugden (1995). In this model, the risk aversion coefficient $\gamma$ is drawn from a normal distribution $N(\mu_\gamma, \sigma_\gamma^2)$ for each decision to be made. The choice is then made to maximize the resulting expected utility function (E(u(z)) with u defined in Equation 1). Estimation of $(\mu_\gamma, \sigma_\gamma^2)$ with maximum likelihood, standard errors are provided in parentheses.

Table A2: Different stochastic specifications (random preferences) for estimates of the utility function in the four experiments.[1]

| | Gain domain | | | | Loss domain | | | |
|---|---|---|---|---|---|---|---|---|
| Parameter | TP | TD | NC | All | TP | TD | NC | All |
| **SWE:** | | | | | | | | |
| $\mu_\gamma$ | 0.943 | 0.977 | | 0.963 | 0.872 | 0.928 | | 0.902 |
| | (0.026) | (0.022) | | (0.017) | (0.018) | (0.020) | | (0.013) |
| $\sigma_\gamma$ | 0.290 | 0.218 | | 0.253 | 0.247 | 0.239 | | 0.245 |
| | (0.046) | (0.028) | | (0.026) | (0.033) | (0.032) | | (0.023) |
| **US:** | | | | | | | | |
| $\mu_\gamma$ | 1.028 | 1.114 | | 1.082 | 0.413 | 0.611 | | 0.518 |
| | (0.046) | (0.042) | | (0.032) | (0.049) | (0.029) | | (0.026) |
| $\sigma_\gamma$ | 0.634 | 0.484 | | 0.561 | 0.764 | 0.750 | | 0.764 |
| | (0.078) | (0.048) | | (0.044) | (0.111) | (0.099) | | (0.075) |
| **AUT I:** | | | | | | | | |
| $\mu_\gamma$ | 0.772 | 0.836 | | 0.808 | 0.801 | 0.854 | | 0.833 |
| | (0.018) | (0.014) | | (0.011) | (0.017) | (0.011) | | (0.009) |
| $\sigma_\gamma$ | 0.322 | 0.260 | | 0.288 | 0.320 | 0.192 | | 0.244 |
| | (0.045) | (0.028) | | (0.025) | (0.043) | (0.017) | | (0.018) |
| **AUT II:** | | | | | | | | |
| $\mu_\gamma$ | 0.916 | 0.870 | 0.942 | 0.907 | 0.853 | 0.914 | 0.849 | 0.874 |
| | (0.027) | (0.016) | (0.022) | (0.012) | (0.020) | (0.018) | (0.017) | (0.011) |
| $\sigma_\gamma$ | 0.409 | 0.264 | 0.313 | 0.323 | 0.344 | 0.267 | 0.300 | 0.306 |
| | (0.040) | (0.018) | (0.025) | (0.015) | (0.029) | (0.019) | (0.022) | (0.013) |

[1] This table corresponds to Table 4 in the main paper. All amounts are converted to US Dollars (using average exchange rates for 2013). Estimates of random preference models with the same parametrization as in Table A1, $N(\mu_\gamma, \sigma_\gamma^2)$, with estimated specifications by experiment. Estimation of $(\mu_\gamma, \sigma_\gamma^2)$ with maximum likelihood, standard errors are provided in parentheses.

## A2. Experimental Instructions (examples of screen shots in Figure A2 and A3)

### A2.1: Experiment 1 and 2: Risk gain/loss experiment (Experiment SWE and Experiment USA)

***Time-Pressure Treatment***

It is important that you carefully read the following instructions. The experiment consists of six parts, each part containing four to seven questions. You will in total have to answer 27 questions.

The first four parts contain questions where you will have to make decisions regarding the allocation of money. At the end of the survey, one of your answers will be drawn at random for actual payment in addition to the $5 (100 SEK) you get for participating. The last two parts contain questions where you will be asked to consider described situations and assess what you believe is right and wrong.

Before each new part a new set of instructions will appear on the screen. It is important that you carefully read these and that you understand the instructions before you start answering the questions.

Try to answer the questions as quickly as possible. In the first 4 parts, you will have a maximum of 7 seconds for each question. A counter on the screen will indicate how much time you have left. If you do not respond within 7 seconds the next question will appear automatically.

NEW SCREEN (RISK GAIN)

In the first 4 questions, you will have to make choices between receiving a sum of money with certainty and participating in a gamble to win a larger amount. If you choose to gamble, we'll flip a coin and if the outcome is heads, you will receive an additional $5 (100 SEK) for your participation, while if the outcome is tails you will get $0 (0 SEK) additionally for your participation. This means that you have 50% chance of winning $5 (100 SEK) additionally if you choose the coin toss.

Remember that you have a maximum of 7 seconds to answer each question. When you press 'next' the following question will appear.

Press 'next' when you are ready to start.

NEW SCREEN

Do you choose $1 (35 SEK) with certainty or to flip a coin for $5 (100 SEK)?
*Time Remaining: 7 seconds*

- $1 (35 SEK) with certainty
- Flip a coin for $5 (100 SEK)

*(Note: we used 35, 40, 45 and 50 SEK as certain gain as monetary variation Experiment 1 (SWE) and $1, $1.5, $2 and $2.5 as certain gain as variation in Experiment 2 (USA)).*

NEW SCREEN (RISK LOSS)

In the next four questions, you will have to make choices between losing a sum of money with certainty and participating in a gamble to avoid losing a larger amount. If you choose to gamble, we'll flip a coin and if the outcome is heads, you will lose the $5 (100 SEK) you get in return for your participation, while if the outcome is tails you will lose $0 (0 SEK) of the money you receive for participating. This means that you have 50% chance of losing your $5 (100 SEK) if you choose coin toss.

Remember that you have a maximum of 7 seconds to answer each question. When you press 'next' the following question will appear.

Press 'next' when you are ready to start.

NEW SCREEN

Do you choose a loss of $1 (35 SEK) with certainty or to flip a coin to avoid a loss of $5 (100 SEK)?

*Time Remaining: 7 seconds*

- Lose $1 (35 SEK) with certainty
- Flip a coin to avoid losing $5 (100 SEK)

*(Note: we used 35, 40, 45 and 50 SEK as certain loss as variation in Experiment 1 (SWE) and $1, $1.5, $2 and $2.5 as certain loss as variation in Experiment 2 (USA)).*

***Time-delay Treatment***

It is important that you carefully read the following instructions. The experiment consists of six parts, each part containing four to seven questions. You will in total have to answer 27 questions.

The first four parts contain questions where you will have to make decisions regarding the allocation of money. At the end of the survey, one of your answers will be drawn at random for actual payment in addition to the $5 (100 SEK) you get for participating. The last two parts contain questions where you will be asked to consider described situations and assess what you believe is right and wrong.

Before each new part a new set of instructions will appear on the screen. It is important that you carefully read these and that you understand the instructions before you start answering the questions.

Think carefully through your response before proceeding. In the first 4 parts, it will take 7 seconds before you can answer each question.

NEW SCREEN

In the first 4 questions, you will have to make choices between receiving a sum of money with certainty and participating in a gamble to win a larger amount. If you choose to gamble, we'll flip a coin and if the outcome is heads, you will receive an additional $5 (100 SEK) for your participation, while if the outcome is tails you will get $0 (0 SEK) additionally for your participation. This means that you have 50% chance of winning $5 (100 SEK) additionally if you choose the coin toss.

Think carefully through your response before proceeding. In the first 4 parts, it will take 7 seconds before you can answer each question.

Press 'next' when you are ready to start.

NEW SCREEN (RISK GAIN)

Do you choose $1 (35 SEK) with certainty or to flip a coin for $5 (100 SEK)?

- $1 (35 SEK) with certainty
- Flip a coin for $5 (100 SEK)

NEW SCREEN (RISK LOSS)

In the next four questions, you will have to make choices between losing a sum of money with certainty and participating in a gamble to avoid losing a larger amount. If you choose to gamble, we'll flip a coin and if the outcome is heads, you will lose the $5 (100 SEK) you get in return for your participation, while if the outcome is tails you will lose $0 (0 SEK) of the money you receive for participating. This means that you have 50% chance of losing your $5 (100 SEK) if you choose coin toss.

Think carefully through your response. It will take 7 seconds before you are able to provide your answer to each question.

Press 'next' when you are ready to start.

NEW SCREEN

Do you choose a loss of $1 (35 SEK) with certainty or to flip a coin to avoid a loss of $5 (100 SEK)?

- Lose $1 (35 SEK) with certainty
- Flip a coin to avoid losing $5 (100 SEK)

## A2.2: Experiment 3: Risk gain/loss experiment (Experiment AUT I)

### *Time-Pressure Treatment*

It is important that you carefully read the following instructions. The experiment consists of six parts, each part containing four to seven questions. You will in total have to answer 27 questions.

The first four parts contain questions where you will have to make decisions regarding the allocation of money. At the end of the survey, one of your answers will be drawn at random for actual payment in addition to the €10 you get for participating. The last two parts contain questions where you will be asked to consider described situations and assess what you believe is right and wrong.

Before each new part a new set of instructions will appear on the screen. It is important that you carefully read these and that you understand the instructions before you start answering the questions.

Try to answer the questions as quickly as possible. In the first 4 parts, you will have a maximum of 7 seconds for each question. A counter on the screen will indicate how much time you have left. If you do not respond within 7 seconds the next question will appear automatically.

NEW SCREEN (RISK GAIN)

In the first 4 questions, you will have to make choices between receiving a sum of money with certainty and participating in a gamble to win a larger amount. If you choose to gamble, we'll flip a coin and if the outcome is heads, you will receive an additional €10 for your participation, while if the outcome is tails you will get €0 additionally for your participation. This means that you have 50% chance of winning €10 additionally if you choose the coin toss.

Press ‘next’ when you are ready to start.

NEW SCREEN

Do you choose €3.5 with certainty or to flip a coin for €10?

*Time Remaining: 7 seconds*

- €3.5 with certainty
- Flip a coin for €10

*(Note: we used €3.5, €4, €4.5 and €5 as certain gain as monetary variation in Experiment 3)*

NEW SCREEN (RISK LOSS)

In the next four questions, you will have to make choices between losing a sum of money with certainty or to participate in a gamble to avoid losing a larger amount. If you choose to gamble, we'll flip a coin and if the outcome is heads, you will lose the €10 you get in return for your participation, while if the outcome is tails you will lose €0 of the money you receive for participating. This means that you have 50% chance of losing your €10 if you choose the coin toss.

Press 'next' when you are ready to start.

NEW SCREEN

Do you choose a loss of €3.5 with certainty or to flip a coin to avoid a loss of €10?

*Time Remaining: 7 seconds*

- Lose €3.5 with certainty
- Flip a coin to avoid losing €10

*(Note: we used €3.5, €4, €4.5 and €5 as certain loss as monetary variation in Experiment 3)*

***Time-delay Treatment***

It is important that you carefully read the following instructions. The experiment consists of six parts, each part containing four to seven questions. You will in total have to answer 27 questions.

The first four parts contain questions where you will have to make decisions regarding the allocation of money. At the end of the survey, one of your answers will be drawn at random for actual payment in addition to the €10 you get for participating. The last two parts contain questions where you will be asked to consider described situations and assess what you believe is right and wrong.

Before each new part a new set of instructions will appear on the screen. It is important that you carefully read these and that you understand the instructions before you start answering the questions.

Think carefully through your response before proceeding. In the first 4 parts, it will take 20 seconds before you can answer each question.

NEW SCREEN

In the first 4 questions, you will have to make choices between receiving a sum of money with certainty and participating in a gamble to win a larger amount. If you choose to gamble, we'll flip a coin and if the outcome is heads, you will receive an additional €10 for your participation, while if the outcome is tails you will get €0 additionally for your participation. This means that you have 50% chance of winning €10 additionally if you choose the coin toss.

Think carefully through your response before proceeding. In the first 4 parts, it will take 20 seconds before you can answer each question.

Press 'next' when you are ready to start.

NEW SCREEN (RISK GAIN)

Do you choose €3.5 with certainty or to flip a coin for €10?

- €3.5 with certainty
- Flip a coin for €10

NEW SCREEN (RISK LOSS)

In the next four questions, you will have to make choices between losing a sum of money with certainty and participating in a gamble to avoid losing a larger amount. If you choose to gamble, we'll flip a coin and if the outcome is heads, you will lose the €10 you get in return for your participation, while if the outcome is tails you will lose €0 of the money you receive for participating. This means that you have 50% chance of losing your €10 if you choose coin toss.

Think carefully through your response. It will take 20 seconds before you are able to provide your answer to each question.

Press 'next' when you are ready to start.

NEW SCREEN

Do you choose a loss of €3.5 with certainty or to flip a coin to avoid a loss of €10?

- Lose €3.5 with certainty
- Flip a coin to avoid losing €10

## A2.3: Experiment 4: Risk gain/loss/loss aversion experiment (Experiment AUT II)

### *General Instructions*

It is important that you carefully read the following instructions. The experiment consists of five parts, each part containing one to five questions. You will in total have to answer 19 questions.

The first three parts contain questions about the allocation of money. One of your answers will be drawn at random for actual payment in addition to the €10 you get for participating. The last two parts contain questions which are not relevant for your payout.

Before each new part a new set of instructions will appear on the screen. It is important that you carefully read the instructions and that you understand them before you start answering the questions. If you have any questions please raise your hand and ask the experimenter before starting a new part. The question will be answered privately.

[PART FOR TIME-PRESSURE]

Try to answer the questions as quickly as possible. In the first four parts, you will have a maximum of 7 seconds for each question. A counter on the screen will indicate how much time you have left (the counter is shown on the screen when there are 6 seconds left and counts down to zero). If you do not respond within 7 seconds the next question will appear automatically. If you fail to answer a question on time and this question is randomly drawn for actual payment, you will lose the €10 you get in return for your participation.

[PART FOR UNCONSTRAINED]

You have no time constraint in answering the questions. When you click ‘OK’ you will proceed to the next question.

[PART FOR TIME-DELAY]

Think carefully through your response before proceeding. In the first four parts, it will take 20 seconds before you can answer each question.

NEW SCREEN

***Instructions for Part 1 (risk gain frame)***

In the first five questions, you will have to make choices between receiving a sum of money with certainty and participating in a lottery to win a larger amount. If you choose the lottery, we will flip a coin and if the outcome is heads, you will receive an additional €10 for your participation, while if the outcome is tails you will get €0 additionally for your participation. This means that you have 50% chance of winning €10 additionally to your participation fee if you choose the coin toss.

[PART FOR TIME-PRESSURE]

Remember that you have a maximum of 7 seconds to answer each question. When you press ‘OK’ the following question will appear.

[PART FOR UNCONSTRAINED]

[empty]

[PART FOR TIME-DELAY]

Think carefully through your response. It will take 20 seconds before you can answer each question. When you press ‘OK’ the following question will appear.

Press ‘OK’ when you are ready to start.

NEW SCREEN

Do you choose €2 with certainty or to flip a coin for €10?

A: Receive €2 with certainty

B: Flip a coin for €10

*(Note: we used €2, €3, €4, €5 and €6 as certain gain as monetary variation in Experiment 4)*

***Instructions Part 2 (risk gain frame)***

In the next five questions, you will have to make choices between losing a sum of money with certainty and participating in a lottery to avoid losing a larger amount. If you choose the lottery, we will flip a coin and if the outcome is heads, you will lose the €10 you get in return for your participation, while if the outcome is tails you will lose €0 of the money you receive for participating. This means that you have a 50% chance of losing your €10 participation fee if you choose the coin toss.

[PART FOR TIME-PRESSURE]

Remember that you have a maximum of 7 seconds to answer each question. When you press ‘OK’ the following question will appear.

[PART FOR UNCONSTRAINED]

[empty]

[PART FOR TIME-DELAY]

Think carefully through your response. It will take 20 seconds before you can answer each question. When you press ‘OK’ the following question will appear.

Press ‘OK’ when you are ready to start.

Do you choose a loss of €2 with certainty or to flip a coin to avoid a loss of €10?

A: Lose €2 with certainty

B: Flip a coin to avoid losing €10

*(Note: we used €2, €3, €4, €5 and €6 as certain loss as monetary variation in Experiment 4)*

***Instructions Part 3 (loss aversion)***

In the next five questions, you will have to decide if you want to participate in a lottery where you can win or lose money. If you choose the lottery, we'll flip a coin and if the outcome is heads, you will receive €8 in addition to your participation fee, while if the outcome is tails you will lose a certain amount of the money you receive for participating. This means that you have a 50% chance of winning additionally to your participation fee and a 50% chance of losing part of it. If you choose not to gamble you will receive €0 in addition to your participation fee.

[PART FOR TIME-PRESSURE]

Remember that you have a maximum of 7 seconds to answer each question. When you press 'OK' the following question will appear.

[PART FOR UNCONSTRAINED]

[empty]

[PART FOR TIME-DELAY]

Think carefully through your response. It will take 20 seconds before you can answer each question. When you press 'OK' the following question will appear.

Press 'OK' when you are ready to start.

Do you choose a payout of zero with certainty or to flip a coin to win €8 or lose €2?

A: €0 with certainty in addition to your participation fee of €10.

B: Flip a coin to win €8 or lose €2.

*(Note: we used €2, €4, €6, €8 and €10 as loss as monetary variation in Option B in Experiment 4)*

***Instructions Part 4 (Jellybeans task)***

On the next screen you will see two bowls. One bowl has 100 jellybeans, and the other has 10 jellybeans. You will be asked to choose one of the two bowls. Please imagine that once you have selected a bowl, it will be placed behind a screen, the experimenter will mix up the jellybeans randomly, and then you will reach around the screen (without looking at the bowl) and select a bean.

Imagine that if you selected a colored bean, you would win €5. Would you prefer to pick from bowl A or bowl B?

[PART FOR TIME-PRESSURE]

Remember that you have a maximum of 7 seconds to answer each question. When you press ‘OK’ the following question will appear.

[PART FOR UNCONSTRAINED]

[empty]

[PART FOR TIME-DELAY]

Think carefully through your response. It will take 20 seconds before you can answer the question.

Press ‘OK’ when you are ready to start.

Which bowl would you prefer if a colored bean wins €5?

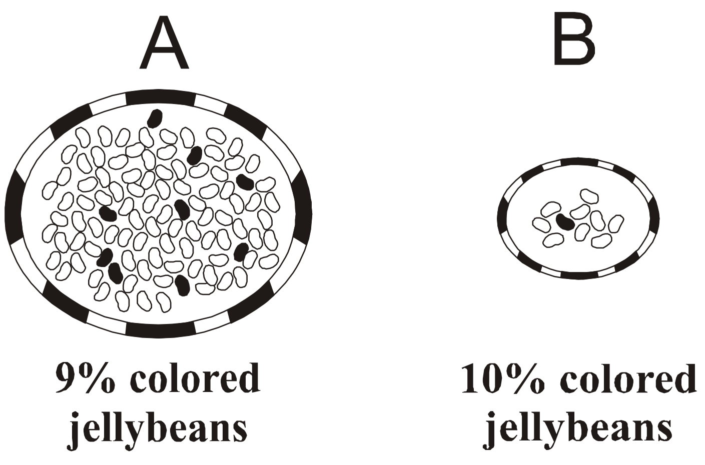

***Instructions Part 5 (CRT)***

Below are three questions that vary in difficulty. Answer as many as you can.

Note that you have no time constraint for answering these questions.

A bat and a ball cost €1.10 in total. The bat costs €1.00 more than the ball.

How much does the ball cost?

____ cents

If it takes 5 machines 5 minutes to make 5 widgets, how long would it take 100 machines to make 100 widgets?

____ minutes

In a lake, there is a patch of lily pads. Every day, the patch doubles in size. If it takes 48 days for the patch to cover the entire lake, how long would it take for the patch to cover half of the lake?

____ days

## A2.4: Screenshots

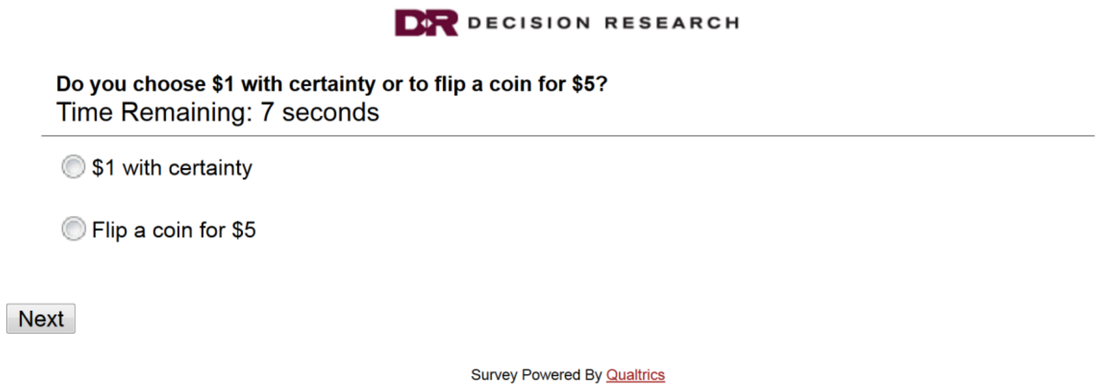


Figure A2. Example screenshot from the decision screen in the time pressure treatment for risk-gains (Experiment USA).

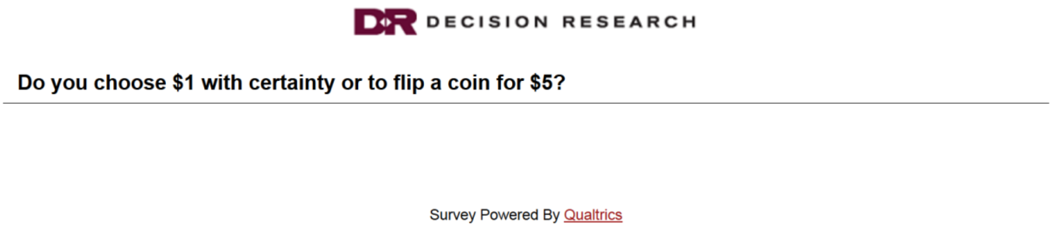


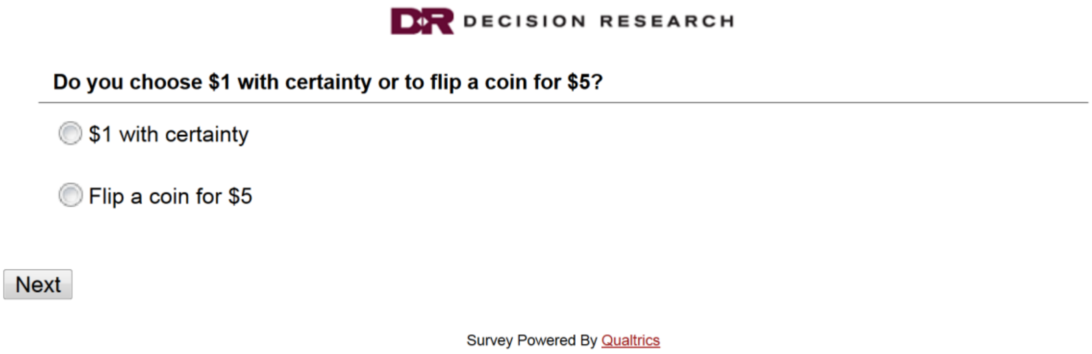


Figure A3. Example screenshots from the decision screen in the time delay treatment for risk-gains (Experiment USA). The top screen shows the time screen during the 7 second time delay and the bottom screen shows the screen after the 7 second time delay (when subjects can enter their answers).

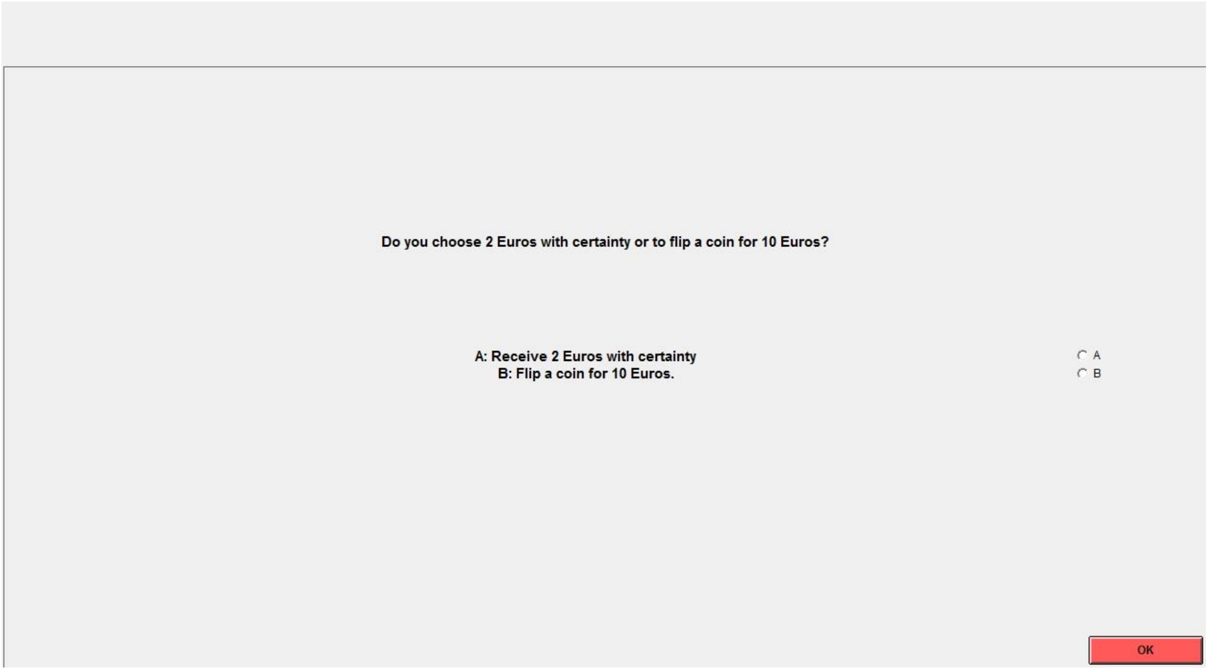


Figure A4. Example screenshot from the decision screen in the treatments for risk-gains in Experiment AUT II. In the time-pressure treatment the remaining decision time is shown on the top right corner of the screen. In the time-delay treatment the OK button and the radio buttons for selecting options A or B appear after 20 seconds.
